# Combined Assessment Findings of High Spleen Index and Fibrosis-4 Index Correlate With Fibrosis Progression in Fontan-Associated Liver Disease: A Retrospective Study

**DOI:** 10.7759/cureus.97976

**Published:** 2025-11-27

**Authors:** Takahiro Fuji, Yumi Otoyama, Masashi Sakaki, Erika Nomura, Ikuya Sugiura, Youko Nakajima, Yuki Ichikawa, Yuu Shimozuma, Manabu Uchikoshi, Hiroaki Kise, Takanari Fujii, Hideshi Tomita, Hitoshi Yoshida

**Affiliations:** 1 Department of Medicine, Division of Gastroenterology, Showa Medical University School of Medicine, Tokyo, JPN; 2 Pediatric Heart Disease and Adult Congenital Heart Disease Center, Showa Medical University, Tokyo, JPN

**Keywords:** fibrosis-4 index (fib-4), fontan-associated liver disease, liver fibrosis, noninvasive assessment, spleen index

## Abstract

Background: The Fontan procedure is a functional palliative surgery for patients with univentricular congenital heart disease. However, Fontan circulation exhibits unique hemodynamics, with chronic elevation of central venous pressure and decreased cardiac output, leading to hepatic venous congestion, reduced portal blood flow, and progressive liver fibrosis, collectively known as Fontan-associated liver disease (FALD). Advanced FALD cases may progress to cirrhosis or hepatocellular carcinoma. Because liver biopsy in FALD carries a risk of bleeding, noninvasive fibrosis assessment methods are needed. This study aimed to analyze the associations among the spleen index (SI), Fibrosis-4 index (FIB-4), and aspartate aminotransferase (AST)-to-platelet (PLT) ratio index (APRI) and ultrasound findings in FALD to examine their utility as noninvasive monitoring indicators.

Methods: This single-center retrospective observational study included 46 post-Fontan patients referred between 2018 and 2024. Data on age, sex, years after surgery, circulatory parameters (central venous pressure and B-type natriuretic peptide), and blood test findings were collected, and FIB-4 and APRI were calculated. SI was calculated as the product of splenic diameter and thickness (cm×cm) on abdominal ultrasound, with SI ≥20 indicative of splenomegaly. Two blinded evaluators independently assessed fine hyperechoic foci. Analyses included the following: (1) comparisons between SI groups (≥20 vs. <20) and between FIB-4 groups (median: 0.682), (2) correlation analysis between SI and FIB-4, and (3) comparison of one-year FIB-4 change (ΔFIB-4) among three risk groups.

Results: The median patient age was 19 years (range: 11-52 years), and the median post-Fontan duration was 14 years (range: 4-40). Splenomegaly was present in 28 patients (61%). Compared with the no splenomegaly group, the splenomegaly group had significantly lower PLT counts and higher FIB-4. Compared with the low FIB-4 group, the high FIB-4 group had significantly higher age and postoperative years, higher B-type natriuretic peptide, lower PLT count, and more frequent fine hyperechoic foci. The SI and FIB-4 scores showed a significant positive correlation (r=0.46; p=0.0013). The FIB-4, but not SI, showed a strong positive correlation with age (r=0.68; p<0.001). Comparisons among the three risk groups showed a significant difference in ΔFIB-4 (p=0.005). Compared with the low-risk group, the high-risk group showed approximately twice the FIB-4 increase (p=0.003), suggesting a threshold effect for fibrosis progression. SI and FIB-4 are noninvasive indicators of liver fibrosis progression in FALD, reflecting different pathophysiological aspects.

Conclusions: SI and FIB-4 are noninvasive indicators of liver fibrosis progression in FALD, reflecting different pathophysiological aspects. SI may reflect the circulatory burden that appears relatively early after surgery, whereas FIB-4 reflects fibrosis accumulation due to aging and chronic congestion. A simultaneously elevated SI and FIB-4 may be associated with rapid fibrosis progression. A combined assessment may be useful for risk stratification and longitudinal monitoring. The relatively low threshold of FIB-4 ≥0.682 may be a practical indicator to detect early FALD-specific fibrosis. Simple monitoring combining splenic assessment by ultrasound and FIB-4 calculation from blood tests can help in the early detection and prognostic evaluation of FALD.

## Introduction

The Fontan procedure is a functional repair surgery for single-ventricle congenital heart disease, and advances in surgical techniques and perioperative management have enabled long-term survival [1,2]. However, Fontan circulation involves a unique hemodynamic state in which systemic venous blood flows directly into the pulmonary artery, resulting in the chronic elevation of central venous pressure and decreased cardiac output. This leads to hepatic venous congestion, reduced portal blood flow, and tissue hypoxia, which interact in combination to cause hepatic parenchymal damage and fibrosis progression [3,4]. This hepatic impairment is called Fontan-associated liver disease (FALD) [3-5]. Patients with FALD initially show fibrosis localized around the central vein (center of the hepatic lobule), but the fibrosis spreads over time, with progression to cirrhosis having been reported [5,6]. Many patients of more than 10 years post-Fontan surgery show fibrotic findings on biopsy, and the degree of progression correlates with postoperative duration [5,7,8].

Accurate assessment of FALD progression is extremely important for prognostic prediction and risk stratification in hepatocellular carcinoma. However, in post-Fontan patients, liver biopsy carries the risk of bleeding due to concomitant anticoagulation therapy and elevated central venous pressure, making it unsuitable for regular, repeated evaluation. Therefore, noninvasive fibrosis assessment methods are required. Transient elastography has recently attracted attention as a method for evaluating fibrosis in FALD, with reports showing that liver stiffness values correlate with central venous pressure and postoperative duration [9,10]. Additionally, hematologic fibrosis markers, such as the Fibrosis-4 index (FIB-4) and aspartate aminotransferase (AST)-to-platelet (PLT) ratio index (APRI), are widely used in chronic liver disease evaluation [11,12]. However, because patients with FALD are predominantly young, the direct application of established cutoff thresholds for metabolic dysfunction-associated steatotic liver disease and hepatitis C virus potentially introduces misclassification, resulting in either the overestimation or the underestimation of the condition [1]. Furthermore, fine hyperechoic foci in the hepatic parenchyma on abdominal ultrasound may reflect mild fibrosis [13]. Hence, their clinical application as a noninvasive, highly reproducible evaluation indicator is being investigated.

Splenomegaly is a classic indicator of portal hypertension and is associated with fibrosis progression and thrombocytopenia in patients with cirrhosis [14,15]. However, reports systematically examining the extent to which the degree of splenomegaly is related to hepatic fibrosis markers and hepatic parenchymal changes in patients with FALD are limited. We conducted this retrospective, single-center observational study. The primary aim of this study was to analyze the correlation between the spleen index (SI), an indicator of splenomegaly, and FIB-4, a marker of hepatic fibrosis, in patients with FALD. Secondarily, we evaluated other fibrosis markers, including APRI and fine hyperechoic foci on ultrasonography, as well as liver dysfunction indicators such as the Model for End-Stage Liver Disease (MELD), MELD-Sodium (MELD-Na), and MELD Excluding International Normalized Ratio (INR) (MELD-XI) scores. Additionally, patient characteristics stratified by FIB-4 values were examined to verify the utility of SI as a noninvasive monitoring indicator in patients with FALD.

## Materials and methods

Study design and population

This single-center retrospective observational study was approved by the Showa Medical University Research Ethics Review Board (approval number: 2023-236-A) and was conducted in accordance with the principles of the Declaration of Helsinki. All data were anonymized prior to analysis. Written informed consent was waived due to the retrospective nature of the study. An opt-out approach was used, with study information provided on the institutional website.

Of the 50 post-Fontan patients referred to our department between 2018 and 2024, 46 patients meeting the following eligibility criteria were included in the analysis: (1) abdominal ultrasound examination, (2) blood tests performed during the same period, and (3) data necessary for evaluation. Among the four excluded patients, two patients had no ultrasound examination, and two patients had asplenia.

Data collection

Data were retrospectively collected from medical records. These included age, sex, Fontan procedure type, postoperative duration, Fontan pressure, B-type natriuretic peptide (BNP; reference <18.4 pg/mL) value, and liver function test parameters (i.e., AST (reference 13-30 U/L), alanine aminotransferase, lactate dehydrogenase, gamma-glutamyl transferase, total bilirubin, albumin, prothrombin time-international normalized ratio (PT-INR) (reference 0.8-1.2), and PLT (reference 15-40×10^4^/μL) count). The FIB-4 and APRI, as noninvasive evaluation indices of hepatic fibrosis, were then calculated. APRI was calculated as $\left[\left(\text{AST/upper limit of normal} \right)/\text{platelet count}(10^{9}/\text{L})\right]\times100$. MELD and MELD-Na scores were calculated using standard formulas based on serum bilirubin, creatinine, INR, and sodium values. All 46 patients had complete data for the primary evaluation items and for calculating the SI and FIB-4.

Imaging protocol

Experienced hepatologists or sonographers performed the abdominal ultrasound examinations using Canon Aplio i800 or Aplio 500 ultrasound systems (Canon Medical Systems, Tokyo, Japan) equipped with convex probes. The Aplio i800 utilized Canon's ultra-wideband convex transducers (i8CX1) with intelligent Dynamic Micro-Slice (iDMS) technology, while the Aplio 500 employed wideband convex transducers. Patients were examined in the supine position after fasting for at least six hours to minimize bowel gas interference. For spleen evaluation, the SI was calculated as the product of the spleen length from the splenic hilum (radius of maximum diameter) and spleen thickness (cm×cm). SI ≥20 cm² was defined as splenomegaly, and SI <20 cm² was defined as no splenomegaly. As there was no established FIB-4 cutoff value for fibrosis evaluation in FALD, this study employed an exploratory approach using the median FIB-4 value (0.682) of the study population to classify patients into two groups and compare their clinical characteristics. To reduce measurement bias, the presence or absence of fine hyperechoic foci in the hepatic parenchyma was independently assessed by two evaluators blinded to patient information. Discordance was resolved through a discussion between the two evaluators.

Statistical analysis

The patients were first classified into two groups based on SI ≥20 and on median FIB-4, and between-group comparisons were performed using the Mann-Whitney U test or Fisher's exact test. Next, the correlations of SI and FIB-4 with age were evaluated using Spearman's rank correlation coefficients.

The annual change in FIB-4 (ΔFIB-4) was calculated using the data of 31 patients (67%) with available follow-up blood test data at one year. The patients were classified into three risk groups based on their baseline SI and FIB-4, and differences in ΔFIB-4 distribution among the groups were determined using the Kruskal-Wallis test. When significant differences were found, the Tukey-Kramer honestly significant difference (HSD) method for multiple comparison testing was performed. All statistical analyses were performed using JMP Student Edition 18 (SAS Institute Inc., Cary, North Carolina, United States). A p-value of <0.05 was considered significant.

Sample size and power

This was an exploratory retrospective study, and an a priori sample size calculation was not performed. All eligible patients (N=46) during the study period were included in the analysis. Post hoc power analysis confirmed that sufficient statistical power (>90%) was obtained for the main correlation observed in this study (SI and FIB-4; r=0.46) under the condition of α=0.05.

## Results

Patient background

The median patient age was 19 years (range: 11-52 years), and the median post-Fontan duration was 14 years (range: 4-40 years). A total of 19 (41%) patients were male, and 28 patients (61%) showed splenomegaly on abdominal ultrasonography. The median FIB-4 and APRI were 0.68 (range: 0.2-3.95) and 1.62 (range: 0.66-5.51), respectively. The background characteristics of the 46 patients are presented in Table 1.

**Table 1:** Baseline patient characteristics (n=46) Data are presented as the median (range).

Characteristic	Value
Age, years	19 (11-52)
Male sex, %	41
Time after Fontan surgery, years	14 (4-40)
Atriopulmonary connection/total cavopulmonary connection	2/44
Platelet count, ×10⁴	15.9 (5.5-30)
Prothrombin time-international normalized ratio	1.31 (0.58-2.26)
Total protein, g/dL	7.3 (5.2-9.4)
Albumin, g/dL	4.6 (2.3-5.4)
Total bilirubin, mg/dL	1.2 (0.5-4.9)
Aspartate aminotransferase, U/L	24.5 (11-65)
Alanine transaminase, U/L	22.5 (7-66)
Lactate dehydrogenase, U/L	176 (126-326)
Gamma-glutamyl transferase, U/L	70 (19-279)
B-type natriuretic peptide, pg/ml	11.4 (5.8-119.9)
Alpha-fetoprotein, ng/mL	3 (1.2-55)
Mac-2 binding protein glycosylation isomer	0.33 (0.11-0.96)
Central venous pressure, mmHg	12 (7-19)
Cardiac index, L/min/m²	2.6 (1.2-4.2)
Saturation of arterial oxygen, %	91 (84-97)
Spleen index, cm²	22 (7-59)
Fine hyperechoic foci, n	24 (52%)
Fibrosis-4 index	0.68 (0.2-3.95)
Aspartate aminotransferase-to-platelet ratio index	1.62 (0.66-5.51)
Model for End-Stage Liver Disease score	10.5 (6.43-17.6)
Model for End-Stage Liver Disease-Sodium score	7 (6-16)
Model for End-Stage Liver Disease Excluding International Normalized Ratio score	10.4 (9.44-17.6)

SI ≥20 correlates with thrombocytopenia and elevated FIB-4

Comparison of clinical characteristics between the SI ≥20 group (n=28) and the SI <20 group (n=18) is shown in Table 2. Age, post-Fontan duration, or hemodynamic parameters showing circulatory dynamics (central venous pressure, cardiac index, and BNP). However, the SI ≥20 group had significantly lower PLT count (p=0.006) and significantly higher FIB-4 (p=0.0496).

**Table 2:** Comparison of clinical parameters according to spleen index Data are presented as median (range) for continuous variables and n (%) for categorical variables. The Mann-Whitney U test was used for continuous variables, and Fisher's exact test was used for categorical variables. Test statistics (U value for Mann-Whitney U test) are presented alongside p-values. *p<0.05 was considered statistically significant.

	SI <20 (no splenomegaly; n=18)	SI ≥20 (with splenomegaly; n=28)	Test statics	P-value
Age, years	17 (12-39)	20 (11-52)	256	0.67
Male sex, %	9 (47)	18 (67)	-	0.19
Time after Fontan surgery, years	14 (4-33)	15 (7-40)	244	0.49
Platelet count, ×10⁴	18.2 (9.4-30)	13.3 (5.5-25.5)	399	0.006*
Prothrombin time-international normalized ratio	1.26 (1.08-2.26)	1.33 (0.58-2.18)	218	0.37
Total protein, g/dL	7.4 (6.3-9.4)	7.3 (5.2-7.8)	313	0.28
Albumin, g/dL	4.6 (4-5.4)	4.6 (2.3-5.2)	251	0.49
Total bilirubin, mg/dL	1.1 (0.7-2.8)	1.3 (0.5-4.9)	249	0.56
Aspartate aminotransferase, U/L	24 (18-40)	24 (11-65)	276	0.99
Alanine transaminase, U/L	25 (14-41)	21 (7-66)	326	0.26
Lactate dehydrogenase, U/L	178 (134-324)	176 (126-326)	257	0.76
Gamma-glutamyl transferase, U/L	66 (30-279)	72 (19-244)	276	>0.99
B-type natriuretic peptide, pg/ml	15.2 (5.8-120)	10.3 (5.8-107)	325	0.18
Alpha-fetoprotein, ng/mL	3 (1.2-6)	3 (2-55)	122	0.36
Mac-2 binding protein glycosylation isomer	0.33 (0.11-0.4)	0.45 (0.17-0.96)	42	0.14
Central venous pressure, mmHg	11.5 (7-17)	13 (10-19)	111	0.12
Cardiac index, L/min/m²	2.6 (1.2-4.2)	2.65 (1.6-3.9)	72	>0.99
Saturation of arterial oxygen, %	91 (84-97)	93 (86-95)	157	0.75
Cirrhotic morphology on ultrasound, n	11 (57%)	20 (74%)	-	0.25
Fine hyperechoic foci, n	9 (50%)	15 (54%)	-	0.49
Fibrosis-4 index	0.59 (0.27-1.09)	0.73 (0.31-3.95)	187	0.0496*
Aspartate aminotransferase-to-platelet ratio index	1.33 (0.7-3.01)	1.72 (0.86-5.51)	194	0.07
Model for End-Stage Liver Disease score	10.1 (7.5-17.6)	11.6 (6.43-17.2)	237	0.39
Model for End-Stage Liver Disease-Sodium score	6 (6-15)	9 (6-16)	179	0.06
Model for End-Stage Liver Disease Excluding International Normalized Ratio score	9.93 (9.44-14.74)	10.8 (9.44-17.6)	235	0.35

FIB-4 is associated with disease duration and markers of liver fibrosis and hepatic dysfunction

The comparison of clinical characteristics between patients with FIB-4 <0.682 and ≥0.682 is shown in Table 3. Compared with the FIB-4 <0.682 group (n=23), the FIB-4 ≥0.682 group (n=23) was significantly older (p=0.004) and had significantly longer post-Fontan duration (p=0.01). Further, in the FIB-4 ≥0.682 group, the PLT count was significantly lower (p<0.001), and the PT-INR was significantly prolonged (p=0.04). With respect to circulatory dynamics parameters, the BNP levels were significantly higher in the FIB-4 ≥0.682 group (p=0.01). For ultrasound findings, in the FIB-4 ≥0.682 group, fine hyperechoic foci representing fibrotic findings were significantly more frequent, and the APRI (p=0.004), MELD score (p=0.01), and MELD-Na score (p=0.04) were significantly higher.

**Table 3:** Comparison of patient characteristics between the high and low Fibrosis-4 index groups Data are presented as median (range) for continuous variables and n (%) for categorical variables. The Mann-Whitney U test was used for continuous variables, and Fisher's exact test was used for categorical variables. Test statistics (U value for Mann-Whitney U test) are presented alongside p-values. *p<0.05 was considered statistically significant.

Variable	Fibrosis-4 index <0.682 (n=23)	Fibrosis-4 index >0.682 (n=23)	Test statistics	P-value
Age, years	17 (12-35)	23 (11-52)	134	0.004*
Male sex, %	8 (35)	12 (52)	146	0.37
Time after Fontan surgery, years	13 (4-26)	19 (7-40)	474	0.01*
Platelet count, ×10⁴	18.8 (13.3-30)	12.6 (5.5-23.4)	474	<0.0001*
Prothrombin time-international normalized ratio	1.21 (0.78-2.26)	1.39 (0.58-2.18)	151	0.04*
Total protein, g/dL	7.3 (3.5-9.4)	7.4 (5.2-8)	254	>0.99
Albumin, g/dL	4.6 (3.5-5.4)	4.6 (2.3-5.2)	268	0.56
Total bilirubin, mg/dL	1.1 (0.5-4.9)	1.3 (0.6-2.9)	232	0.48
Aspartate aminotransferase, U/L	26 (18-40)	24 (11-65)	311	0.32
Alanine transaminase, U/L	25 (14-66)	17 (7-50)	381	0.01*
Lactate dehydrogenase, U/L	177 (137-324)	176 (126-326)	254	0.59
Gamma-glutamyl transferase, U/L	62 (23-279)	75 (19-204)	231	0.46
B-type natriuretic peptide, pg/ml	0.68 (0.43-1.02)	0.69 (0.38-0.98)	136	0.01*
Alpha-fetoprotein, ng/mL	10.1 (5.8-110)	21.6 (5.8-119.9)	131	0.35
Mac-2 binding protein glycosylation isomer	3 (1.2-8)	3.5 (2-55)	1	0.15
Central venous pressure, mmHg	0.315 (0.17-0.38)	0.46 (0.11-0.96)	141	0.38
Cardiac index, L/min/m²	12 (9-17)	12 (7-19)	55	0.22
Saturation of arterial oxygen, %	2.44 (1.2-3.4)	2.8 (0.6-4.2)	211	0.18
Cirrhotic morphology on ultrasound, n	92 (84-97)	90.5 (86-94)	-	0.12
Spleen index	19 (7-37)	26 (13-59)	163	0.03*
Fine hyperechoic foci, n	13 (57%)	18 (78%)	-	0.03*
Aspartate aminotransferase-to-platelet ratio index	1.43 (0.7-3.01)	1.89 (0.86-5.51)	132	0.004*
Model for End-Stage Liver Disease score	9.44 (6.43-17.6)	12.2 (7.6-17.2)	144	0.01*
Model for End-Stage Liver Disease-Sodium score	6 (6-15)	8 (6-16)	114	0.04*
Model for End-Stage Liver Disease Excluding International Normalized Ratio score	9.93 (9.44-17.6)	10.8 (9.44-14.9)	235	0.5

SI positively correlates with FIB-4

**Figure 1:**
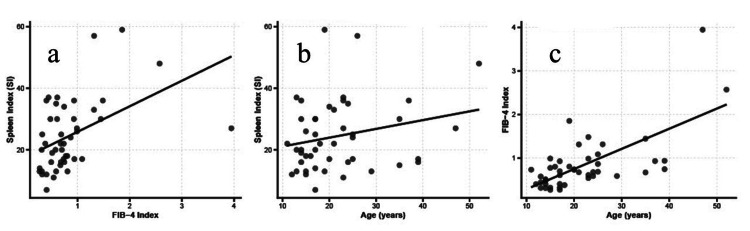
SI positively correlates with FIB-4 but not with age-dependent progression (a) Scatter plot showing the correlation between FIB-4 and SI in Fontan patients. The black line represents the linear regression line (R²=0.213; p=0.001), indicating a significant positive correlation. (b) Scatter plot showing the correlation between SI and age in Fontan patients. The black line represents the linear regression line (R²=0.054; p=0.122), indicating no significant correlation. (c) Scatter plot showing the correlation between FIB-4 and age in Fontan patients. The black line represents the linear regression line (R²=0.457; p<0.001), indicating a significant positive correlation. Correlation relationships are evaluated using Spearman's rank correlation coefficient. FIB-4: Fibrosis-4 index; SI: spleen index

Correlation analysis of SI and FIB-4 as continuous variables revealed a significant positive correlation (R²=0.213; p=0.0013; Figure 1a). The regression equation was SI=17.56+8.29×FIB-4, indicating that SI increased by approximately 8.3 for every 1-unit increase in FIB-4.

FIB-4, but not SI, shows a significant age-dependent increase

Given that FALD progresses with aging and longer post-Fontan duration, a correlation analysis of age with SI and FIB-4 was performed. Linear regression analysis showed that SI demonstrated a slight positive trend with age, but this did not reach statistical significance (R²=0.05; p=0.12; Figure 1b). This indicates that splenomegaly occurs relatively early in the post-Fontan period and does not necessarily progress linearly with age. In contrast, the FIB-4 showed a significant positive moderate correlation with age (R²=0.457; p<0.001; Figure 1c). This suggests that hepatic fibrosis assessed using FIB-4 progresses with age in post-Fontan patients.

Simultaneous elevation of SI and FIB-4 shows a threshold effect on annual fibrosis progression

**Figure 2:**
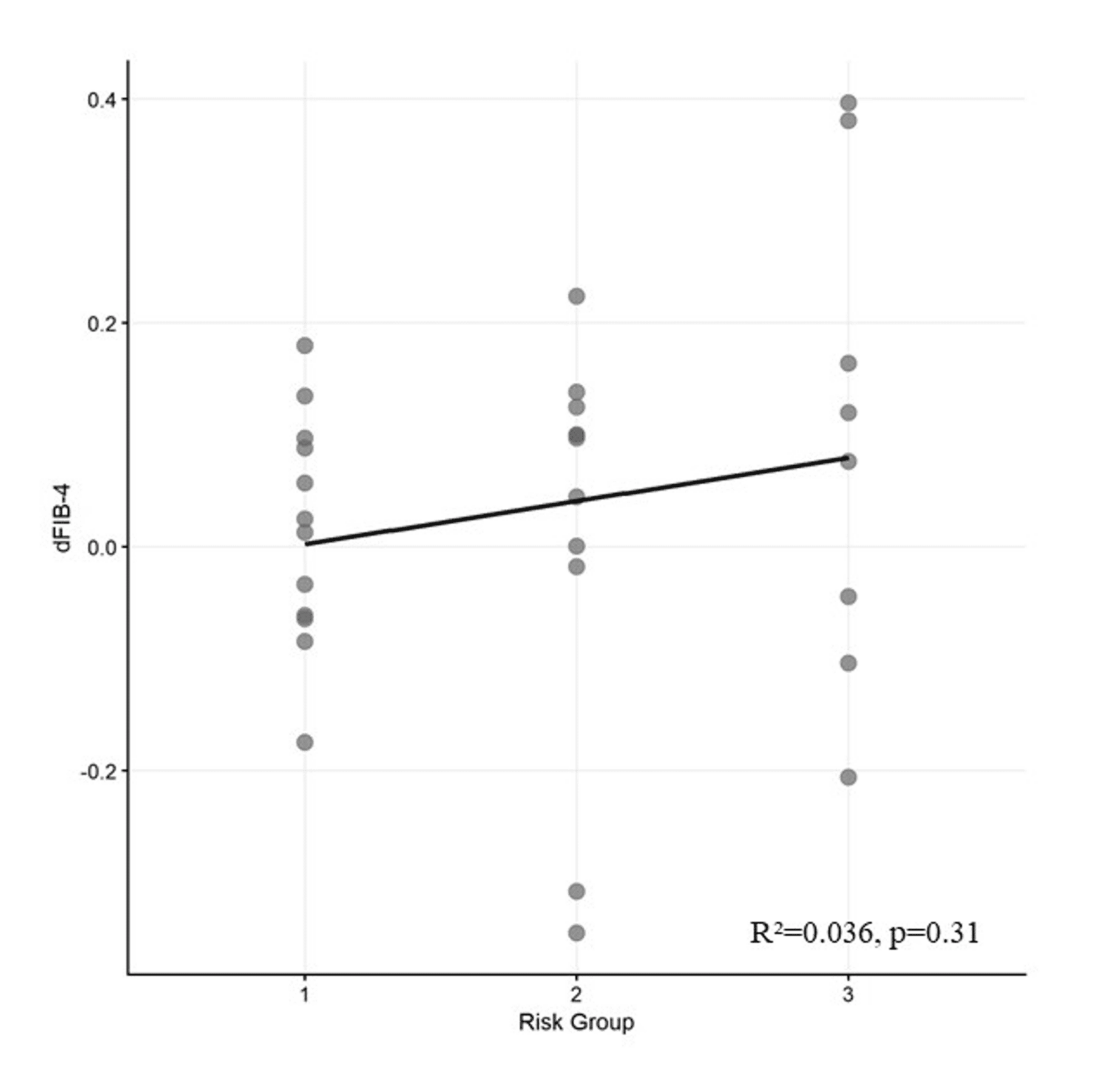
Linear regression analysis showing the relationship between risk group and ΔFIB-4 Scatter plot showing the ΔFIB-4 distribution across the three risk groups. Risk groups: 1=low risk (SI <20 and FIB-4 <0.682), 2=intermediate risk (either elevated), and 3=high risk (both elevated). Each dot represents a single individual. Although no significant linear trend is observed (R²=0.036; p=0.31), the Kruskal-Wallis test reveals significant differences among groups (χ²=10.45; p=0.005), with the high-risk group showing significantly higher ΔFIB-4 than the low-risk group (p=0.003; Tukey-Kramer honestly significant difference test). This pattern suggests a threshold effect rather than continuous linear progression. ΔFIB-4: change in FIB-4 over one year

Risk stratification was performed based on baseline SI and FIB-4, and the relationship with one-year FIB-4 change (ΔFIB-4) was examined (Table 4 and Figure 2). A total of 11, 11, and nine patients belonged to the low-, intermediate-, and high-risk groups, respectively. ΔFIB-4 was significantly different among the three groups (low-risk group (0.52±0.34) vs. intermediate-risk group (0.76±0.34) vs. high-risk group (1.08±0.34); Kruskal-Wallis test; χ²=10.45; p=0.005). In multiple comparison analysis (Tukey-Kramer HSD test), the high-risk group showed significantly higher ΔFIB-4 compared to the low-risk group (mean difference: 0.55; 95% confidence interval: 0.21-0.89; p=0.003). This result indicates that the rate of hepatic fibrosis progression over one year in the high-risk group was approximately twice that in the low-risk group.

**Table 4:** Comparison of annual FIB-4 changes among the risk groups Data are presented as mean±SD. Statistical comparison among the three groups was performed using the Kruskal-Wallis test (χ²=10.45; p=0.005), followed by the Tukey-Kramer honestly significant difference test for post hoc pairwise comparisons. *p<0.05 was considered statistically significant vs. the low-risk group. ΔFIB-4: change in FIB-4 over one year; FIB-4: Fibrosis-4 index; SD: standard deviation; SI: spleen index

Risk group	n	ΔFIB-4 (mean±SD)	Rank score	P-value
Low risk (SI <20 and FIB-4 <0.682)	11	0.52±0.34	9.82	Reference
Intermediate risk (SI ≥20 or FIB-4 ≥0.682)	11	0.76±0.34	16.45	0.26
High risk (SI ≥20, FIB-4 ≥0.682)	9	1.08±0.34	23.00	0.003*

ΔFIB-4 in the intermediate-risk group showed intermediate values between both groups, but no statistically significant differences were found between the low-risk (p=0.26) and high-risk (p=0.11) groups. The linear trend test showed no significant trend (p=0.85). This result suggests the existence of a threshold effect whereby hepatic fibrosis progression markedly accelerates only when both the SI and FIB-4 indicators are simultaneously elevated, rather than the risk increasing in a continuous stepwise manner. This means that, when only one indicator is elevated, the impact on hepatic fibrosis progression is limited. The composite elevation of both indicators is a potentially important predictor of hepatic fibrosis progression.

## Discussion

The present study aimed to evaluate the utility of noninvasive markers, including SI using abdominal ultrasound and FIB-4, for monitoring disease progression in patients with FALD. Our findings demonstrate that patients with splenomegaly (SI ≥20) showed significantly lower PLT counts and higher FIB-4 values compared to those without splenomegaly. Furthermore, patients with elevated FIB-4 (≥0.682) showed a higher frequency of fine hyperechoic foci in the hepatic parenchyma. Fine hyperechoic findings have been previously reported as early imaging findings reflecting mild fibrosis and centrilobular congestive changes [13]. Hence, FIB-4 ≥0.682, although a relatively low cutoff value, may be a clinically useful threshold reflecting such mild fibrosis stages in the population with FALD. These findings indicate that SI serves as a useful indicator reflecting portal hypertension through hypersplenism, while FIB-4 captures hepatic fibrosis progression. Therefore, the combination of SI and FIB-4 may provide comprehensive information about disease progression in patients with FALD.

In Fontan circulation, systemic venous blood flows into the pulmonary circulation without passing through the right heart, resulting in the chronic elevation of central venous pressure. Consequently, hepatic venous congestion persists, and fibrosis progresses over time [3,4]. Furthermore, portal hypertension and splenomegaly appear [4,5]. The current study found a significant positive correlation between SI and FIB-4, indicating a close relation between splenomegaly due to portal hypertension and the progression of hepatic fibrosis. Meanwhile, the FIB-4 (R²=0.457; p<0.001), but not SI (R²=0.05; p=0.12), significantly correlated with age. The slightly positive correlation of SI with age indicates that splenomegaly appears relatively early post-Fontan and subsequently does not increase linearly in proportion to age. Collectively, these results suggest that splenomegaly may be an indicator of circulatory burden occurring relatively early postoperatively, with limited subsequent age-related changes. In contrast, FIB-4 may more strongly reflect chronic congestion and fibrosis progression over the long term. Therefore, SI and FIB-4 are complementary indicators capturing different disease stages of FALD, and the combined use of both may enable the noninvasive assessment of circulatory burden and fibrosis progression in patients with FALD.

Our risk stratification based on baseline SI and FIB-4 revealed the largest increase in one-year FIB-4 (ΔFIB-4) in the high-risk group (SI ≥20 and FIB-4 ≥0.682), with a progression rate approximately twice that of the low-risk group (p=0.003). In the intermediate-risk group (only either SI or FIB-4 elevated), ΔFIB-4 showed intermediate values; however, no significant difference was observed. These results support that when either SI or FIB-4 alone is elevated, fibrosis progression is limited. However, when both are simultaneously elevated, fibrosis tends to progress more markedly. This suggests that a combined assessment of splenomegaly and fibrosis markers may be useful to accurately capture FALD progression. However, it should be noted that FIB-4 is an indicator influenced by nonspecific factors, such as age and PLT count, and its relationship with fibrosis in FALD remains conflicting. In the current study, the frequency of fine hyperechoic findings associated with early mild fibrosis in FALD was significantly higher in the FIB-4 ≥0.682 group than in the FIB-4 <0.682 group, suggesting that FIB-4 may partially reflect the degree of fibrosis progression. FIB-4 and APRI are noninvasive fibrosis markers widely used for estimating the fibrosis stage in chronic hepatitis and non-alcoholic fatty liver disease. Their simplicity and reproducibility have led to attempts to apply them to FALD [9,10]. Because FIB-4 is age-dependent, it is necessary to set a cutoff value that is appropriate for the pathological condition of patients with FALD [7]. Emamaullee et al. reported that FIB-4 significantly correlated with bridging fibrosis in 106 Fontan patients (FIB-4: 0.6±0.4 vs. 0.4±0.2; p<0.01 (AUC: 0.69)) [16]. Similarly, Jarasvaraparn et al. demonstrated that FIB-4 significantly correlated with bridging fibrosis or cirrhosis on liver biopsy in 66 Fontan patients (0.64 vs. 0.32; p=0.02) [17]. The cutoff value of FIB-4 ≥0.682 used in the current study likely reflects the unique pathophysiology of FALD that differs from that of adult chronic liver disease. Although quantitative methods (e.g., magnetic resonance elastography and T1/T2 mapping) are being increasingly introduced, considering cost and implementation limitations [18], simple monitoring by ultrasound examination and blood tests is realistic in actual clinical practice.

This study has limitations. The study was retrospective in nature and included a small population. In addition, pathological histological verification was lacking, making it impossible to directly prove the extent to which fibrosis markers and imaging findings accurately reflected actual fibrosis progression. Furthermore, although multivariate analysis was performed using variables that showed significant differences between the splenomegaly groups (PLT count and FIB-4), no independent predictors were identified, likely due to the small sample size and collinearity between these variables. Thus, the possibility that the observed associations were influenced by other factors could not be completely excluded. Thus, the possibility that the observed associations were influenced by other factors could not be completely excluded. Based on cross-sectional analysis, it is unclear whether splenomegaly or FIB-4 elevation causally preceded the other. Future studies involving longitudinal follow-up of temporal changes through a longitudinal design and comprehensive evaluation, including pathological and hemodynamic indices, are warranted. Given that the results were also obtained from a single center, there may be facility-specific characteristics reflected in patient backgrounds and treatment policies, limiting the generalizability of the results. Therefore, further verification through multicenter collaborative studies is required.

## Conclusions

In patients with FALD, SI and FIB-4 are significantly correlated, and both may be useful for assessing fibrosis progression. Particularly, patients with simultaneously elevated SI and FIB-4 tend to have more profound fibrosis progression. Additionally, the higher frequency of fine hyperechoic findings in the high FIB-4 group than in the low FIB-4 group suggests that FIB-4 may reflect mild fibrosis and congestive changes in FALD. Collectively, these results suggest that the combination of SI and FIB-4 may enable the noninvasive assessment of circulatory burden and fibrosis progression in patients with FALD, potentially contributing to early disease detection and prognosis prediction. Future longitudinal studies combining pathological and hemodynamic indices are required.
